# Influence of chronic stress on the mechanism of the cytotoxic system in common carp (*Cyprinus* *carpio*)

**DOI:** 10.1111/imm.13345

**Published:** 2021-05-31

**Authors:** Mazal Shimon‐Hophy, Ramy R. Avtalion

**Affiliations:** ^1^ Laboratory of Comparative Immunology and Genetics The Mina and Everard Goodman Faculty of Life Sciences Bar‐Ilan University Ramat‐Gan Israel

**Keywords:** acute stress regulation, chronic stress regulation, cytotoxic downregulation, IL‐8 regulation, proinflammatory

## Abstract

Aquaculture conditions expose fish to internal and environmental stressors that increase their susceptibility to morbidity and mortality. The brain accumulates stress signals and processes them according to the intensity, frequency duration and type of stress, recruiting several brain functions to activate the autonomic or limbic system. Triggering the autonomic system causes the rapid release of catecholamines, such as adrenaline and noradrenaline, into circulation from chromaffin cells in the head kidney. Catecholamines trigger blood cells to release proinflammatory and regulatory cytokines to cope with acute stress. Activation of the limbic axis stimulates the dorsolateral and dorsomedial pallium to process emotions, memory, behaviour and the activation of preoptic nucleus‐pituitary gland‐interrenal cells in the head kidney, releasing glucocorticoids, such as cortisol to the bloodstream. Glucocorticoids cause downregulation of various immune system functions depending on the duration, intensity and type of chronic stress. As stress persists, most immune functions, with the exception of cytotoxic functions, overcome these effects and return to homeostasis. The deterioration of cytotoxic functions during chronic stress appears to be responsible for increased morbidity and mortality.

## Graphical abstract

Chronic stress suppresses the immune system and especially the metabolism of components in the cytotoxic system, and the proliferation and maturation of cells essential for the body's protection against disease agents. This may be the reason for the increase in morbidity in chronic stressful situations.

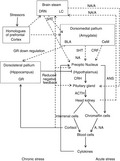

**Abbreviations**
5‐HT: 5‐hydroxytryptamine
A: adrenaline
ACTH: adrenocorticotropic hormone
ANS: autonomic nervous system
AR: adrenergic receptor
CD: cluster of differentiation
CNS: central nervous system
CRF: corticotropin‐releasing factor
CRH: corticotropin‐releasing hormone
DRN: dorsal raphe nucleus
FoxP3: forkhead box P3
GABA: gamma‐aminobutyric acid
GR: glucocorticoid receptor
HPA: hypothalamus‐pituitary‐adrenal gland
HPI: hypothalamus‐pituitary‐interrenal cells
IFN: interferon
IgM: immunoglobulin M
IL: interleukin
MAIT: mucosal‐associated invariant T cell
MCH: melanin‐concentrating hormone
MR: mineralocorticoid receptor
NA: noradrenaline
NCC: nonspecific cytotoxic cell
NCCRP1: nonspecific cytotoxic cell receptor protein 1
TGF: transforming growth factor
Th1: T helper 1 cell
TNF: tumour necrosis factor

## INTRODUCTION

Aquaculture conditions are often exposed to various stressors. Stressors can be a consequence of elevated rearing densities [1], suboptimal water quality, decreased dissolved oxygen and elevated carbon dioxide (CO_2_) levels [2, 3], thermal fluctuations [4, 5], diet [6], presence of enemies and pathogens [7, 8, 9, 10, 11], and transportation, sorting, handling and confinement stresses [6, 12, 13, 14].

Stressors were reported to reduce hippocampal (dorsolateral pallium in teleost) volume [15, 16, 17] and, as a result, to impact memory and learning [18, 19, 20]. The amygdala—in particular, the basolateral amygdala (dorsomedial pallium in teleost)—increases dendritic length and spine density, and as a result, there are changes in emotions [21, 22]. Furthermore, stress exaggerates adverse effects, such as shrinking of the thymus and spleen or other lymphatic organs, changes in the number and distribution of leucocytes, or appearance of bleeding or ulcers that increase susceptibility to morbidity and mortality [23]. Stressors have negative impacts on different physiological responses associated with growth, nutrition, reproduction and immune responses [3, 24, 25, 26, 27, 28, 29]. Understanding and monitoring the biological mechanisms underlying stress responses in fish may alleviate the harmful effects of stress through selective breeding and changes in management practices, resulting in improved animal welfare and production efficiency.

This review will summarize the processes that mainly regulate chronic stress and influence immune system functions. These processes severely impair the cytotoxic functions in the immune system, and as result, they have implications for morbidity, mortality and efficiency of production in aquaculture. The evaluation of stress's influence on the immune system will be based primarily on previous studies conducted in our laboratory.

## BRAIN REGULATION OF STRESS

The stress mechanism is still far from explaining the detailed molecular processes and the exact brain structures that participate in stress regulation, but it is known that, unlike mammals, the fish's telencephalon lacks a cortex but possesses telencephalon cortical‐like functions, as reported in several fish species [30]. The fish's telencephalon contains several distinct neuronal populations that have been characterized as functional homologues to mammalian forebrain areas. For example, the dorsomedial and dorsolateral pallium have been characterized as functional homologues to the mammalian basolateral amygdala and hippocampus, respectively, and are implicated in stimulus salience, memory and learning [31, 32, 33]. Furthermore, the ventral part of the telencephalon was reported to be functionally homologous to the lateral septum [31, 33], which is very important in the regulation of emotional reactivity and goal‐oriented behaviour[34, 35, 36].

**FIGURE 1:**
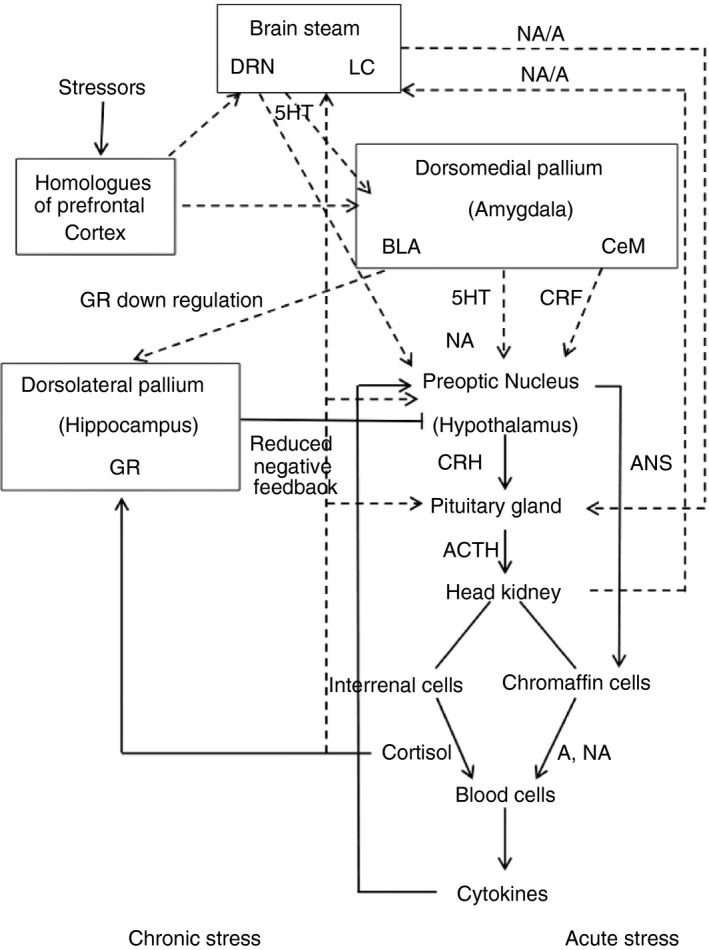
Putative regulation of stress in common carp. Acute stress usually activates the sympathetic neurons in the autonomic nervous system (ANS), which, in turn, activates the chromaffin cells of the head kidney to release catecholamines, such as adrenaline (A) and noradrenaline (NA). Catecholamines bind to their receptors in the blood cells and promote the production of specific cytokines. Chronic stress activates the axis of hypothalamus‐pituitary‐interrenal cells of the head kidney (HPI) and promotes the release of corticotropin‐releasing hormone (CRH) from the hypothalamus. This activates the pituitary gland to release adrenocorticotropic hormone (ACTH) into the bloodstream, which then allows the secretion of cortisol from the interrenal cells. Cortisol binds to its receptors in blood cells and, as a result, various processes take place according to the intensity and duration of stress. Similarly, cortisol in the feedback process regulates hypothalamic, hippocampal and locus coeruleus (LC) activity. Stressor stimuli from various brain areas, such as prefrontal cortex‐like formation, LC and dorsal raphe nucleus (DRN), stimulate the amygdala to elicit the proper activation of the HPI axis and different body functions. The amygdala facilitates the release of NA, corticotropin‐releasing factor (CRF) and 5‐hydroxytryptamine (5‐HT) from the hypothalamus. The amygdala likely attenuates the negative feedback exerted by glucocorticoids by reducing hippocampal glucocorticoid receptors (GR), thus facilitating HPI axis activation

Mammalian studies have found that the brain accumulates external and internal signals of stress, processes them and recruits several neuronal circuits to maintain physiological integrity [37]. The intensity, frequency, duration and type of stress will evoke autonomic stress response or limbic circuits, such as the prefrontal cortex, amygdala, hippocampus, paraventricular nucleus of the hypothalamus and the nucleus accumbens [38, 39]. The amygdala (dorsomedial pallium in teleost) functions like a command centre that processes emotions and sends stress signals to the hypothalamus (preoptic nucleus in teleost), while the hypothalamus works as a command centre that communicates through other parts of the body, such as the autonomic nervous system and the hypothalamus‐pituitary‐adrenal/head kidney axis to control functions, such as breathing, blood pressure, heart rate and the immune system [40] (Figure 1). Excessive or inadequate basal activity and responsiveness of this system might impair development, growth and body composition, and lead to a host of behavioural and somatic pathological conditions [41].

## AUTONOMIC NERVOUS SYSTEM (ANS) REGULATION OF STRESS

In mammals and teleost fish, immune organs are innervated by sympathetic neurons. In fish, sympathetic innervation of lymphoid tissue has been found in the spleen of coho salmon (*Oncorhynchus kisutch*), where nerve fibres are associated with vasculature and melanomacrophage centres [42]. Moreover, immune cells express receptors for stress hormones and neurotransmitters, including adrenergic receptors (AR). Mammalian innate immune cells express both α‐ and β‐ARs subtypes, while exclusive expression of ARs of the β2 subtype has been found on T and B lymphocytes [43].

In mammals, lymphoid organs are innervated by sympathetic and parasympathetic nerve fibres [44, 45] whose activation stimulates or inhibits the immune response. Furthermore, leucocytes express both cholinergic and adrenergic receptors [46]. However, little is known about the fish cholinergic system versus the fish adrenergic system, which is predominant in the stress response (Figure 1). Catecholamine receptors are present on the immune cells of teleost fish [47], and many lymphoid tissues receive sympathetic innervation. For example, in coho or silver salmon (*Onchorhynchus kisutch*), the spleen is highly innervated by adrenergic fibres in the vasculature and parenchyma [42]. Several radio‐ligand binding experiments have demonstrated the presence of β‐ARs (b‐AR) in the anterior kidney, spleen and peritoneal leucocytes of goldfish (*Carassius auratus*) [48], and in the head kidney and spleen leucocytes of the American catfish (*Ictalurus punctatus*) [49]. The influence of sympathetic innervations on the immune system of teleost fish is exerted through the binding of adrenaline and noradrenaline to their functional adrenoceptors, α‐AR (a‐AR) and b‐AR, which are present in immune system cells [47]. Catecholamines inhibit the innate and acquired immune response in various species of teleosts through b‐AR activation. However, a‐AR stimulation leads to the production of antibodies [47, 50, 51, 52] The adrenoceptor b2a‐AR mRNA is constitutively expressed in the brain, especially in the preoptic nucleus (homologous to the mammalian hypothalamus) and immune organs. During the in vivo inflammatory response, b2a‐AR expression is upregulated in the peritoneal leucocytes. Additionally, adrenaline inhibits the expression of proinflammatory cytokines, chemokines and their receptors in fish phagocytes cultured in vitro [53]. Adrenaline may influence the inflammatory response via direct regulation of leucocyte migration or apoptosis during zymosan‐induced peritoneal inflammation in the common carp [54]. Similar to the ANS responses in mammals [55], these responses in fish can be influenced by the immune system through cytokines produced by glial cells (e.g. astrocytes) in the central nervous system (CNS), which modulates neuroendocrine responses. The ANS response can also be altered by peripheral signals that gain access to the CNS through circumventricular organs, which are structures without blood‐brain barriers [56]. Nevertheless, it was recently confirmed that adrenaline behaves in different ways in different teleost species. While adrenaline did not modulate the expression of immune‐related genes in rainbow trout (*Oncorhynchus mykiss*) head kidney primary cell culture, adrenaline enhanced the expression of interleukin 1β (IL‐1b) and transforming growth factor‐β1 (TGF‐b1) stimulated by inactivated *V. anguillarum* in sea bream (*Sparus aurata*), and the effect was diminished by propranolol [57]. Conversely, catecholamine secretion from teleost chromaffin cells in the head kidney is regulated by a host of cholinergic and non‐cholinergic pathways that ensure sufficient redundancy and flexibility in the secretion process to permit synchronized responses to a myriad of stressors [58].

## HYPOTHALAMUS‐PITUITARY‐INTERRENAL (HPI) AXIS REGULATION OF STRESS

In mammals, the hypothalamus‐pituitary‐adrenal (HPA) axis is modulated by extra‐hypothalamic limbic structures, particularly the hippocampus and the amygdala [59, 60]. While hippocampal neurons exert an inhibitory effect on the activation of the axis, amygdala activity exerts a significant facilitating effect [59]. The amygdala has two direct efferent connections and one indirect efferent connection with the hypothalamus: (1) the stria terminalis directly connects the amygdala with the preoptic area in the hypothalamus; (2) the ventral pathway directly connects the central amygdala and basolateral amygdala with the hypothalamus [61]. An indirect pathway consists of projections from the central amygdala to the bed nucleus of the stria terminalis, the efferents of which retro‐project to corticotropin‐releasing factor (CRF) cells in the paraventricular nucleus of the hypothalamus [62]. In teleosts, the mechanism of stress regulation in the HPI axis is still obscure; however, when stress signals are perceived, the hypothalamic region of the nucleus preopticus responds by releasing corticotropin‐releasing hormone (CRH) into the pituitary. This signal is received by CRH receptor subtype 1 (CRH‐R1) on pituitary corticotropes from the pars distalis. The binding of CRH with its receptor stimulates adrenocorticotropic hormone (ACTH) release into circulation [63, 64]. ACTH stimulates the production and release of the main glucocorticoid cortisol from the head kidney's interrenal cells [65] (Figure 1).

Cortisol exerts its effect on target cells by binding to the cytosolic glucocorticoid receptor (GR) [66]. The cortisol‐GR complex translocates into the nucleus, where it binds to responsive glucocorticoid elements and modifies gene expression [67]. As in mammals, both the GR and the mineralocorticoid receptor (MR) can bind cortisol [68]. In contrast to mammals, fish have duplicate GR genes (GR1 and GR2) that are translated into functional proteins [67]. GR1 also exists in two variants: GR1a and GR1b [69, 70]. Thus, there are four receptors capable of binding cortisol in fish: GR1a, GR1b, GR2 and MR. However, their ability to induce downstream gene activation depends on the cortisol concentration [71]. The CRF signal is mediated by at least two receptors (CRFR1 and CRFR2). CRFR1 has been reported to mediate HPI axis activation, whereas CRFR2 contributes to the expression of several behavioural and physiological reactions in response to stress [65, 72]. Moreover, similar to the process with mammals, 5‐hydroxytryptamine (5‐HT) in teleosts influences hypothalamic CRF release, where the 5‐HT receptor type 1A plays a central role in the regulation of the HPI axis [73, 74, 75, 76]. Additionally, the HPI axis is under feedback control by cortisol through the MR and GR in the hypothalamus and pituitary [77, 78, 79]. Studies suggest the presence of interactions between HPI and limbic functions in the teleost telencephalon [30, 80]. Moreover, associations found between telencephalic 5‐HT and HPI axis activities [30, 74, 76, 81, 82, 83] support similar involvement of this section of the brain in HPI axis regulation, as observed in mammals [84].

Glucocorticoids regulate multiple aspects of immune defences in mammals and influence the secretion of proinflammatory and anti‐inflammatory cytokines [85]. Similarly, cortisol receptors have been identified and described in fish immune cells, and cortisol affects the immune response in common carp (*Cyprinus carpio*) [70, 71], rainbow trout (*Oncorhynchus kisutch*) and gilthead sea bream (*Sparus aurata*) [86]. Cortisol influences the secretion of cytokines from leucocytes, and these cytokines regulate the HPI axis activity in response [87]. Additionally, cortisol inhibits proliferation and induces apoptosis in lymphocytes of the blood, head kidney, spleen, and thymus [88]. This process is dependent on the GR and RU486 (mifepristone), a specific GR blocker, preventing these cortisol processes [89]. In mammals, it has been reported that chronic or acute administration of dexamethasone, a potent GR agonist, can cause a significant neurotransmission imbalance between glutamate and gamma‐aminobutyric acid (GABA) via upregulation of GABAergic neurons and downregulation of glutamatergic neurons in the amygdala, and, consequently, cortisol regulates stress‐induced emotions [90]. The main function of ACTH in fish is the regulation of cortisol production in the head kidney's interrenal cells [65]. In rainbow trout (*Oncorhynchus mykiss*), mifepristone use reduces stress‐induced cortisol secretion by reducing hypothalamic CRH mRNA expression [91]. The corticotropic action of CRH can be avoided through the administration of the non‐selective antagonist of the CRH receptor [92]. An additional hypothalamic factor is the melanin‐concentrating hormone (MCH), a strong inhibitor of CRH‐stimulated ACTH secretion [93, 94]. Rainbow trout (*Oncorhynchus mykiss*) that acclimated to abundant light had higher MCH and ACTH levels and lower cortisol levels in plasma, unlike fish acclimated to a dark environment [95, 96]. MCH is a peptide that mediates colour changes in teleost fish (an antagonist of the alpha‐melanocyte‐stimulating hormone a‐MSH) [97], and its plasma levels are modified under stress conditions. However, hypothalamic MCH regulates food intake and energy balance in mammals [98] and goldfish (*Carassius auratus*) [99]. Nonetheless, the effect of MCH is significantly lower than the effect of CRH on food intake and energy balance in fish under stress conditions.

## INFLUENCE OF STRESS ON THE IMMUNE SYSTEM

Studying the effect of stress on the immune system is challenging due to the variable responses in different individual carps. Therefore, following up changes in cytokines and leucocytes levels in peripheral blood during stress treatments was preferred over sampling their levels in the spleen, kidney, head kidney and liver. Monitoring the blood enables changes in each carp to be ascertained without killing the specimen [100]. A systematic study revealed which function of the carp immune system was most affected by hypoxic stress and how the duration of stress influences the expression of these functions. Acute stress enhances the fast pathway that activates the sympathetic nervous system to release catecholamines, such as adrenaline and noradrenaline, from chromaffin cells in the head kidney, and the released catecholamines bind to their receptors in leucocytes [101, 102]. As a result, the proinflammatory function (IL‐1b, IL‐6 and tumour necrotic factor α (TNFa)) is upregulated and, at the same time, the activity of regulatory function (TGFb and IL‐10) is upregulated, probably in order to return proinflammatory activity to homeostasis [100, 103]. Chronic stress activates the hypothalamus‐pituitary‐interrenal cell axis and, as a result, interrenal cells in the head kidney mainly release cortisol [65]. The cortisol binds to its receptors in leucocytes and promotes different processes in the leucocytes [65, 67].

The results of monitoring the influence of chronic hypoxic stress on immune activity in the common carp peripheral blood leucocytes are shown in Table 1, and these results reveal a downregulation of regulatory (IL‐10, TGFb, forkhead box P3 (FoxP3)), proinflammatory (IL‐1β, IL‐6), and inflammatory (IL‐17) functions until the second week of chronic stress. However, in the third week, their change in levels overcame and returned to homeostasis [100]. TNFa levels did not change during hypoxic stress treatments (Table 1), but TNFa behaved slightly differently in chronic cortisol implants in rainbow trout (*Oncorhynchus mykiss*) for five days [104]. The chronic cortisol treatment showed results similar to those in acute hypoxic stress (Table 1). In contrast, the main impaired functions, even after 22 days of chronic stress [100, 105], were as follows: (1) cytotoxic mediators, such as interferon (IFN)‐γ2b, Fas ligand (FasL), NK lysin and granzyme; (2) IL‐12 and Tbet, which are responsible for Th1 cell proliferation and maturation, which mediates host defence against intracellular pathogens [106, 107, 108]; and (3) IL‐8, which attracts leucocytes to the infection site [109]. IL‐8, which was downregulated during the 22‐day chronic stress period, can explain the macrophage/neutrophil/leucocyte mobilization decline in different body compartments, as shown by Wojtaszek and colleagues [110].

**TABLE 1:** Changes in the levels of mRNA components that represent different functions in the immune system of common carp following stress

Cytokines	Con	AS	CSW1	CSW2	CSW3
IL1b	1·00 ± 0·12	5·15 ± 0·67*	1·42 ± 0·17	0·06 ± 0·02*	2·69 ± 0·60*
IL6	1·00 ± 0·18	1·47 ± 0·28*	1·43 ± 0·79	0·16 ± 0·11	1·16 ± 0·20
TNFa	1·00 ± 0·14	3·73 ± 0·27*	1·29 ± 0·17	0·61 ± 0·06	0·79 ± 0·10
IFNg2b	1·00 ± 0·12	1·4 ± 0·19	0·00 ± 0·00*	0·00 ± 0·00*	0·00 ± 0·00*
C3s	1·00 ± 0·8	0·79 ± 0·21	18·44 ± 9·11	4·43 ± 1·91	10·66 ± 5·36
IgM	1·00 ± 0·12	1·35 ± 0·16	1·67 ± 0·22	1·51 ± 0·10	1·99 ± 0·30
IL10	1·00 ± 0·15	3·01 ± 0·34*	0·35 ± 0·07*	0·0006 ± 0·0001*	0·51 ± 0·07
FoxP3	1·00 ± 0·14	2·51 ± 0·73	0·27 ± 0·04*	0·0021 ± 0·0004*	0·80 ± 0·14
TGFb	1·00 ± 0·14	1·98 ± 0·21*	0·99 ± 0·13	0·0027 ± 0·0004*	3·63 ± 0·48
IL8	1·00 ± 0·1 3	0·81 ± 0·08	0·18 ± 0·03*	0·0016 ± 0·0003*	0·30 ± 0·07*
CD95	1·00 ± 0·28	1·14 ± 0·17	1·78 ± 0·4	3·38 ± 0·87*	2·34 ± 0·45*
FasL	1·00 ± 0·17	1·00 ± 0·19	0·83 ± 0·18	0·47 ± 0·1*	0·23 ± 0·08*
granzyme	1·00 ± 0·39	0·45 ± 0·11	0·81 ± 0·28	0·50 ± 0·14	0·26 ± 0·06*
NKlyzin	1·00 ± 0·61	0·26 ± 0·07*	0·35 ± 0·12	0·25 ± 0·06*	0·30 ± 0·07*
NILT1	1·00 ± 0·81	1·77 ± 0·57	1·50 ± 0·50	1·39 ± 0·63	0·56 ± 0·26
NILT2	1·00 ± 0·31	1·56 ± 0·71*	1·32 ± 0·59	1·78 ± 0·60	0·77 ± 0·23
IL12b	1·00 ± 0·18		0·06 ± 0·12*	1·04 ± 0·99*	0·00003 ± 0·00006*
Tbet	1·00 ± 0·41	0·90 ± 0·27	0·52 ± 0·11	0·72 ± 0·18	0·29 ± 0·13*
STAT4	1·00 ± 0·63	3·16 ± 0·45*	1·03 ± 0·31	1·51 ± 0·55*	0·75 ± 0·28
CXCR3	1·00 ± 0·38	0·83 ± 0·21*	0·84 ± 0·34	0·80 ± 0·23	0·44 ± 0·32

Note:

The above results are aggregated from references.[100, 105].

Abbreviations: AS, acute stress; Con, control; CSW1, chronic stress after 8 days; CSW2, chronic stress after 15 days; CSW3, chronic stress after 22 days.

* *P* ≤ 0·05.

In contrast to the sharp decrease in the level of cytotoxic cytokines following chronic stress, nonspecific cytotoxic receptor protein 1 (NCCRP1) levels increased sharply. It has been confirmed that the NCCRP1, which was previously related to a marker of nonspecific cytotoxic cells (NCC) [111] and as a variant of NK cells in teleosts, is not a marker of any cell type, but is abundant in γδT, mucosal‐associated invariant T (MAIT), T carp lymphocytes and even in thrombocytes [112]. Further study will clarify what role it plays in stress processes.

Chronic administration of cortisol (simulating chronic stress) decreased the relative expression of IFNa‐1, heat shock proteins 70 (HSP70) and 90 (HSP90), **s**erum amyloid A protein and glucocorticoid receptors in *Salmo salar* [113]. Macrophage cell lines revealed the inhibition of chemotaxis, phagocytosis, and respiratory burst activity in goldfish (*Carassius auratus*) [114]. These chronic administrations of cortisol strengthened the downregulation of cytotoxic functions by chronic stress (Table 1).

Innate function (immunoglobulin M (IgM) and complement C3s mRNA) (Table 1) was not significantly affected during acute or chronic hypoxic stress treatments [100], chronic confinement stress events of juvenile Eurasian perch (*Perca fluviatilis*) [115] or high stocking density of *Eleginops maclovinus* [29]. These results contradicted findings regarding husbandry, confinement and crowding‐induced stresses [4, 116, 117, 118]. Presumably, these differences among the results are attributable to the presence of modulators that regulate IgM humoral activity [119]. Similarly, C3s mRNA showed no significant changes in either acute or chronic stresses, although its levels fluctuated throughout the chronic stress period (Table 1). These results differ from haemolytic findings [9, 120]; however, they are consistent with reported hypoxia and cortisol‐induced stress results [121, 122].

**FIGURE 2:**
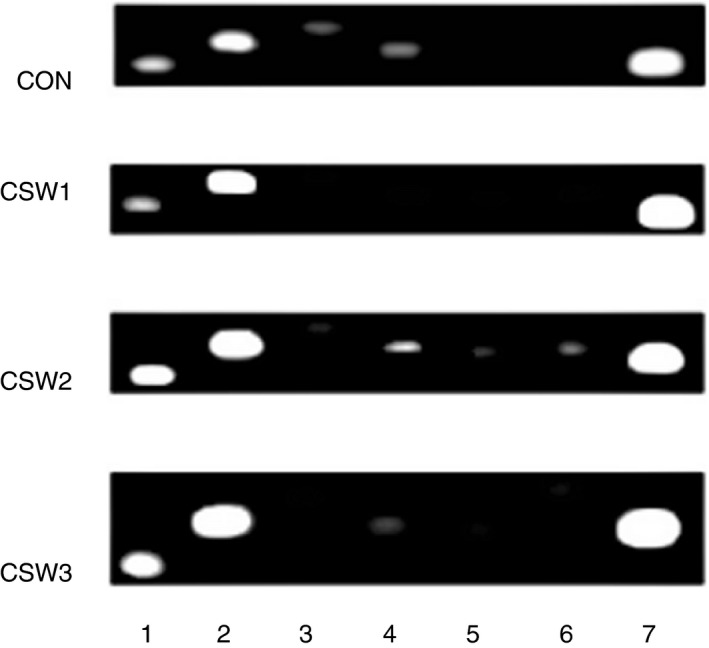
The distribution of leucocyte types in common carp peripheral blood following stress treatments. Cell markers were produced from mixed 1000 ng cDNA of eight fish by PCR amplification and loaded on 1·3% agarose gel with TBE (Tris/Borate/EDTA) running solution. **(**1) T cell (CD3‐TCRε), (2) γδT cells (TCRγδ), (3) CD4, (4) CD8, (5) NK cells (CD56), (6) macrophages/monocytes (CD209) and (7) NCCRP1

Stress‐influenced functions revealed the deterioration of cytotoxic activity and cytokines regulating Th1 proliferation (Table 1), but what about the other leucocytes? Studies of leucocyte levels by fluorescence‐activated cell sorting (FACS) and by mRNA levels of cell markers revealed a decrease in the levels of like‐B, like‐plasma, macrophages and CD4 (Th1) cells (Table 2 and Figure 2) [100]. These results are consistent with others’ findings of a decrease in leucocyte numbers in *Oncorhynchus mykiss* [123], the suppression of phagocytic and lymphocyte proliferative activities in *Platichthys flesus* and *Solea senegalensis* [124], and the apoptosis of B cells in *Cyprinus carpio* [125]. However, we cannot be certain if stress also caused MAIT cell deterioration [112], because of contrasting microscopic results; therefore, there is a need to further study these cells. *In vitro* studies confirm the above‐mentioned results, revealing that cortisol treatments had the following effects: (1) decreased the phagocytosis of head kidney cells from tilapia (*Oreochromis niloticus x* *O. aureus*), common carp (*Cyprinus carpio*), and silver sea bream (*Sparus sarba*) [126]; (2) inhibited the pro‐oxidative activity of leucocytes from the head kidneys of golden sea bream (*Sparus aurata*) [127]; (3) inhibited the proliferation of monocyte/macrophage cell lines from rainbow trout (*Oncorhynchus mykiss*) [128]; and (4) induced programmed cell death (apoptosis) of macrophages from silver sea bream (*Sparus sarba*) and Atlantic salmon (*Salmo salar*) [129].

**TABLE 2:** Changes in the cell types following stress treatments in peripheral blood leucocytes of common carp

Treatment	Cell type	Con	AS	CSW1	CSW2	CSW3
mRNA levels	CD4	1·00 ± 0·41	0·92 ± 0·37	0·37 ± 0·12*	0·38 ± 0·11*	0·14 ± 0·06*
	CD8a	1·00 ± 0·44	1·16 ± 0·56	0·85 ± 0·16	1·18 ± 0·48	0·38 ± 0·14
	T (TCRε)	1·00 ± 0·23	0·52 ± 0·06*	0·38 ± 0·06*	0·49 ± 0·09*	0·41 ± 0·06*
	γδT(TCRγδ)	1·00 ± 0·13	1·29 ± 0·35	1·70 ± 0·40*	1·29 ± 0·23	0·67 ± 0·22
Cell per cent	Monocytes/macrophages	1·05 ± 0·09	1·01 ± 0·32	0·30 ± 0·06*	0·26 ± 0·09*	0·21 ± 0·05*
	B‐like cells	8·50 ± 1·69	4·28 ± 0·95*	3·86 ± 1·40	1·34 ± 0·37*	1·38 ± 0·17*
	Plasma‐like cells	4·86 ± 2·52	3·76 ± 0·76	2·54 ± 0·70	1·64 ± 0·42*	0·93 ± 0·25*

Abbreviations: AS, acute stress; Con, control; CSW1, chronic stress during a 1‐week period; CSW2, chronic stress during a 2‐week period; CSW3, chronic stress during a 3‐week period; results aggregated from references [100, 105].

* *P* ≤ 0·05.

Cluster of differentiation 8 (CD8), NK and γδT cells (Table 2 and Figure 2) did not show any decrease corresponding to that of cytotoxic cytokines, although they are known for the high production of IFNγ, FasL, granzyme and NK lysin [130, 131, 132, 133]. Figure 2 has not yet been published, but is shown here to emphasize that the changes in cytokines shown in Table 1 are not the result of cell destruction but rather of their metabolism impairment. Moreover, γδT cells are the most numerous cells in carp leucocytes (Figure 2) and are thought to be the greatest producers of IFNγ [134, 135]. However, their cell amounts do not decrease following chronic stress or the decrease in cytotoxic cytokine levels. This indicates that chronic stress suppresses cytotoxic cytokine metabolism and the proliferation of Th1, macrophages/monocytes and plasma cells. Consequently, this suppression may explain the increased susceptibility to diseases resulting from chronic stress [3, 116, 120, 136].

**FIGURE 3:**
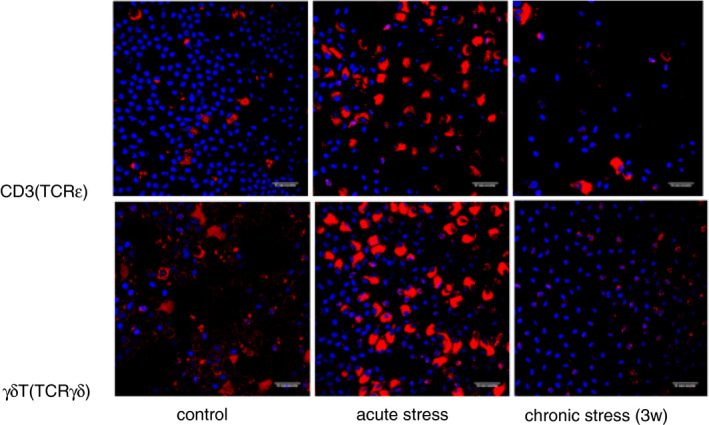
The difference in the cell volume of T and γδT cells in common carp peripheral blood following stress treatments. ‘3w’ denotes chronic stress after 22 days. Scale bar—10 μm. Results were adapted from reference [112]

The decrease or increase in metabolism was shown in the volume of the cells (Figure 3). During acute stress responses, when the metabolism of proinflammatory and regulatory cytokines was upregulated, cell volume increased up to three times (according to measurements of the cell area), while during chronic stress responses, the cell volume of γδT cells decreased up to three times following three‐week periods of chronic stress. Figure 3 reinforces the perception that chronic stress mainly impairs the metabolism of cytotoxic cytokines.

## SUMMARY

The continued sustainability of the aquaculture industry depends on its profitability. Stress is considered to be a major factor contributing to poor health in cultured fish. Studying the influence of stress on the immune system enables us to recommend tools to manage fish sensitivity, morbidity, and mortality in fish ponds.

The mechanisms of processes regulating the immune system during stress have not been fully elucidated in mammals and are even more unclear in fish. Little is known about the specific aetiological pathways that lead from a triggering stressor to the development of a specific pathological phenotype, or the interactions between neurotransmitters, such as NA, 5HT, GABA and glutamate.

Despite the clear involvement of brain structures, such as the amygdala, hippocampus and HPI axis, it remains unclear how these structures cause various pathological disorders, as well as how they cause different responders to respond differently to the same stress stimuli. Previous studies on different stress responses have reported similar changes with respect to neurotransmitter activity, neuroplastic changes and alterations in amygdalar and HPI function, suggesting that these properties are common and that phenotypic specificity is rooted in upstream mechanisms.

Recent studies indicate that the brain accumulates and processes stress signals and activates several brain structures to maintain physiological integrity. The intensity, duration and type of stress evoke autonomic system or limbic circuits. The autonomic system immediately responds to acute stress and stimulates chromaffin cells in the head kidney to release proinflammatory and regulatory cytokines. The limbic structures tend to respond slowly to chronic stress; the limbic homologs of the amygdala and hippocampus accumulate signals from different brain areas to process emotions and the memory of stress, and activate the HPI axis and other body functions, such as blood pressure, heart rate and energy accumulation. The HPI axis stimulates interrenal cells in the head kidney to release glucocorticoid hormones, such as cortisol, to the bloodstream. Glucocorticoids deteriorate cytotoxic activity, resulting in the downregulation of cytokines involved in cytotoxic activity and the downregulation of cell proliferation as well as cells involved in phagocytosis, antibody production and Th1. The downregulation of cytotoxic activity is critical for disease resistance and unwanted cell elimination. Therefore, further study of the mechanistic processes of stress regulation is required to reduce fish morbidity and mortality.

## CONFLICT OF INTEREST

The authors have no conflicts of interest to report.

## AUTHOR CONTRIBUTIONS

Mazal Shimon‐Hophy edited and wrote the manuscript. Ramy R. Avtalion gave the final approval of the version to be submitted.
